# Dispositional Traits, Characteristic Adaptations, and Narrative Identity Reconstructions in Individuals With Depersonalization and Derealization

**DOI:** 10.1111/jopy.12976

**Published:** 2024-10-17

**Authors:** Emanuele Fino, Thalia Jemmett‐Skinner, Richard Evans‐Miller, Joe Perkins, Mohammed Malik, Martin Robinson, Gwendalyn Webb

**Affiliations:** ^1^ School of Psychology Queen's University Belfast Belfast UK; ^2^ NTU Psychology Nottingham Trent University Nottingham UK; ^3^ Unreal Bristol UK

**Keywords:** depersonalization, derealization, identity, individual differences, personality

## Abstract

**Introduction:**

Depersonalization and derealization disorder (DPDR) is a debilitating condition. To date, little was known about the role of personality structure and of perceived social support and loneliness in DPDR.

**Methods:**

Three studies investigated, respectively: (i) broadband personality traits (five‐factor model), maladaptive trait domains (PID‐5), and perceived support and loneliness in individuals with self‐reported DPDR (*N* = 160) versus a general population sample (*N* = 303), using network modeling; (ii) structure and interconnectivity of personality, perceived support and loneliness, and DPDR traits (frequency/duration) in individuals with self‐reported DPDR (*N* = 160); (iii) characteristic adaptations and narrative identities in individuals with self‐reported DPDR (*N* = 19), using thematic analysis.

**Results:**

Study 1 found between‐samples differences across several traits, especially psychoticism and negative affect. Differences in networks' global centrality, but not structures or edges, were also found. The graphical model in Study 2 showed a community of dissociative tendencies including DPDR traits and psychoticism. Study 3 highlighted the development of DPDR as a key life transition for those experiencing it, with narratives focusing on feelings of poor agency, isolation, and a disrupted sense of self.

**Conclusions:**

Individual differences in personality characterize DPDR, especially in psychoticism. Implications for theory and research are discussed.

## 1 Introduction

The Diagnostic and Statistical Manual of Mental Disorders, version 5 (DSM‐5), categorizes depersonalization and derealization (DPDR) as a dissociative disorder, denoting a breakdown and compartmentalization of psychobiological functions that normally work in unison (American Psychiatric Association [APA] 2013). DPDR manifests through an integrated and related set of symptoms, primarily: (i) a feeling of perceptual alteration of surroundings and the external world, as if these were “unreal” or dream‐like (derealization, DR), experienced continually despite intact reality testing (World Health Organization [WHO] 2024); (ii) the often accompanying feeling of being disconnected from one's own thoughts, mind, and/or body, or feeling “like a robot,” for example, observing one's own perceptions from outside the self, while questioning their ownership (depersonalization, DP) (Baker et al. 2003; Medford et al. 2005).

DPDR is comorbid with several psychopathological conditions, including anxiety disorders, depression, obsessive‐compulsive tendencies, and other dissociative syndromes (for a review, see Sierra 2009). This challenges the estimation of its prevalence across psychiatric populations, whereas the prevalence of DPDR in the general population is estimated to be about 1% (Yang et al. 2023). However, this figure may underestimate the actual prevalence of the condition, due to a combination of factors, importantly, the difficulty experienced by individuals with DPDR to verbalize their symptoms (Medford et al. 2005) and the delayed and inadequate assessments that are frequently reported by those experiencing DPDR (Murphy 2023).

### 1.1 The Role of Individual Differences in Depersonalization and Derealization

Individual differences play a role in the development and experience of DPDR. Wolf et al. (2023) found in individuals with dissociative post‐traumatic stress disorder that DP was associated with altered connectivity of the hippocampal region and deteriorated self‐monitoring capacity. These results indicate alterations at the levels of sensory integration, spatial representation, and the experience of stress of individuals, possible mechanisms of DPDR‐related post‐traumatic experiences, in line with evidence from previous neuroimaging studies about psychedelic drug use‐induced DPDR (Carhart‐Harris et al. 2014). However, other studies pointed out the role of individual differences in the development of DPDR, even in the absence of traumatic experiences. For example, Aardema et al. (2010) found that tendency to absorption is associated with a greater likelihood of experiencing DP, after immersion in a virtual reality task. Similarly, Soffer‐Dudek (2023) has recently hypothesized a role for obsessive‐compulsive symptoms and a tendency to absorption in the appearance and intensification of dissociative symptoms. Furthermore, resting‐state fMRI data showed links between DPDR‐related perceptual dysregulation and a disrupted intero‐exteroceptive integration function, manifesting through compartmentalization, detachment from reality, and structural dissociation of personality, including changes in one's sense of self and identity (Scalabrini et al. 2020). Notwithstanding the increasing corpus of evidence that highlights individual differences in the onset, frequency, and severity of DPDR, there are still many unknowns about the structure of personality of those affected by the condition. In particular, little is known about dispositional traits, characteristic adaptations, and narrative identity reconstructions in individuals with DPDR, a gap that the current research aims to address.

### 1.2 Dispositional Traits and DPDR

Ciaunica et al. (2022) have recently described the experience of DPDR in terms of a distinctive way to engage with the world “with two mutually exclusive but equally plausible hypotheses. (1) First, a hypothesis that the best explanation for all the evidence at hand is that ‘I am an embodied perceiver, and I am in control of my perceptual processing’. (2) The alternative hypothesis is that ‘I am an embodied perceiver, but I am not in control of my perceptual processing’” (p. 7). It must be noted that such a definition lends itself to important considerations on the dispositional nature of the altered perception that characterizes DPDR. For example, previous research in personality psychology evidenced individual variation in experiential permeability, namely, a maladaptive personality trait that reflects individual differences in regulating inner perceptions and outer reality interactions (Piedmont et al. 2009). For this reason, understanding and mapping the personality structure and differences in broadband FFM traits and maladaptive trait domains of individuals with DPDR is warranted, potentially shedding light on the individual differences that might predispose to and/or intensify the experience of DPDR.

Extant literature on personality and DPDR is sparse. Few studies have approached the analysis of the associations between DPDR and personality structure, of which, the five‐factor model (FFM; McCrae and John 1992) is considered as one of the most established representations. The FFM posits a hierarchical organization of human personality in five primary factors, namely: neuroticism (negative emotionality and tendency to anxiety and depression), extraversion (sociability, assertiveness, energy), openness to experience (intellectual curiosity, originality, creativity), agreeableness (kindness, soft‐heartedness, cooperativeness), and conscientiousness (discipline, organization, persistence). These dispositional traits present stable and consistent patterns of thought, feeling, and behavior, which are known to play a key role in mental health and wellbeing (Wang et al. 2018), including influencing individuals' perceptions of social support and loneliness (Swickert, Hittner, and Foster 2010; Buecker et al. 2020).

Evidence indicates the possible role of a premorbid personality structure in DPDR, that is, individual differences in personality that may predispose people to mental (ill‐)health, facilitating and/or sustaining the development and experience of DPDR (Aardema et al. 2010; Scalabrini et al. 2020; Soffer‐Dudek 2023). In particular research has shown relations between dissociative symptoms and neuroticism (Kwapil, Wrobel, and Pope 2002), the latter being a known correlate of several psychopathological outcomes. Moreover, Kwapil, Wrobel, and Pope (2002) found an independent factor of dissociative experiences within a six‐factor solution that replicated and expanded the original FFM. A study on individual vulnerability to virtual reality‐induced DP found a correlation between neuroticism and DP scores, but no significant associations between DP and personality (Peckmann et al. 2022). Another study used Cloninger, Svrakic, and Przybeck's (1993) psychobiological model to investigate variations in temperamental traits between panic disorders traits with/without comorbid DP, finding that individuals with DP scored lower on self‐directedness and higher on self‐transcendence than those without DP.

Moreover, self‐directedness and self‐transcendence were found to be associated with PID‐5 psychoticism (Hemmati et al. 2021), one of the five trait domains from the DSM‐5 model of maladaptive personality (PID‐5; Krueger et al. 2012). This is a hierarchical model of personality dysfunction that identifies 25 lower order facets loaded on by five higher order maladaptive trait domains, namely: negative affectivity (emotional lability, anxiousness, relational insecurity), detachment (withdrawal, anhedonia, intimacy avoidance), antagonism (manipulativeness, deceitfulness, grandiosity), disinhibition (irresponsibility, impulsivity, distractibility), and psychoticism (unusual beliefs and experiences, eccentricity, perceptual dysregulation). These represent maladaptive counterparts of the FFM traits (Krueger et al. 2012). Individuals scoring highly in PID‐5 trait domains are likely to present forms of psychopathology, including personality disorders and maladaptive behavior (Krueger et al. 2012).

Among the PID‐5 trait domains, psychoticism is of particular interest for DPDR. Indeed, this maladaptive trait domain is characterized by tendencies to bizarre or unconventional patterns of thinking and perceptual/cognitive dysregulation that are common across personality disorders and dissociative experiences (Watson, Clark, and Chmielewski 2008). Soffer‐Dudek (2023) noted that dysregulated perception (characteristic of psychoticism) is associated with temporal lobe abnormalities, which in turn, are present in obsessive‐compulsive symptoms (Ertekin et al. 2009). For this reason, they hypothesized a relation between obsessive‐compulsive personality and DPDR, in the same vein as Torch (1978), which would explain the constant self‐monitoring, repetitive and intrusive thoughts often observed in DPDR (Medford et al. 2005; Soffer‐Dudek 2023). Regarding bonding and interpersonal connectivity, DPDR has been found to be associated with introversion, loss of interest and of motivation to pursue relationships and attachments, subjective feelings of loneliness, isolation, and avoidance of social and group situations (Michal et al. 2006).

### 1.3 Characteristic Adaptations and Narrative Identity Reconstructions

In DPDR, chronic alterations to experiences within one's self and the environment trigger constant and rigid questioning about the perception of being in control of one's own thoughts, feelings, and behavior (Ciaunica et al. 2022; Medford et al. 2005). This impacts individuals' ability to design and implement effective strategies to cope with symptoms and derive a coherent sense of meaning and purpose in life, in a world that is perceived as constantly at risk of turning “unreal (Perkins, 2021).” Therefore, understanding individuals with DPDR's *characteristic adaptations* and *narrative identity reconstructions* (McAdams 2013) would help gauge a greater understanding of how individuals experience, adapt to, and make sense of their symptoms, with important implications for theory and intervention. These features are conceptualized by McAdams (2013) in their theory of human personality. The theory defines personality as a three‐layered construct, respectively, being characterized by: (i) individuals' *dispositional traits*, which define personality structure and map individuals' relatively stable and consistent patterns of thoughts, feelings, and behaviors; (ii) *characteristic adaptations*, that is, beliefs, motives, and coping strategies that help individuals adjust to circumstances and face adversities (McAdams and Olson 2010); and (iii) *narrative identity*, that is, the sense of self and identity that individuals reconstruct through narrating and reflecting on their own story, a continuously evolving subjective framework of self/others perceptions, personal agency, and social interactions. This model has so far informed a diverse range of research areas, including psychopathology, where understanding the role of beliefs and coping strategies against symptoms helps shed light on the mechanisms of recovery, which in turn, supports the further development of theory and clinical applications (Weststrate, Jayawickreme, and Wrzus 2022). However, regarding DPDR, a systematic and integrative exploration of dispositional traits, characteristic adaptations, and narrative identity is missing.

DPDR patients' reports (e.g., see Perkins 2021) typically suggest impairments in *agency* and *communion*, two central themes of narrative identity, respectively, relevant to the need to master one's environment and to cooperate with others (McAdams 2018). Agency reflects the sense of ownership, empowerment, and achievement that individuals communicate through their narratives, that is, beliefs in their ability to influence the course of their own and others' lives through their actions. Communion refers to the extent and quality of interpersonal connections and attachments (e.g., family, close relationships, group affiliations), their role in the experience of and in coping with adversities, and the feelings of support versus isolation and loneliness that people derive from them. Exploring narrative reconstructions of lifelong agency and communion in individuals with DPDR may therefore further help understand the process of integrating the altered perception associated with DPDR symptoms within a cohesive sense of self and of the world, clarifying barriers and facilitators to symptoms' management and recovery, which are vital to clinical intervention and practice. These narratives are expressed in the form of themes, characters, and events that help individuals shape an understanding of how they “came to be and where he or she is going in life” (McAdams and Olson 2010, 527).

### 1.4 The Present Research

To the best of the authors' knowledge, no previous research has approached the study of DPDR primarily from the perspective of personality psychology. The present research aims to address this gap through three studies, respectively aiming to: (i) compare mean differences, structure, and conditional interconnectivity of broadband FFM and maladaptive PID‐5 trait domains and perceived support/loneliness in individuals with DPDR versus individuals from the general population; (ii) model, examine, and evaluate a network of DPDR traits in relation to the same model tested in Study 1, this time, limited to individuals with self‐reported DPDR; and (iii) explore characteristic adaptations and narrative identities of individuals living with the condition, aiming to expand and integrate the understanding of the role of personality structure and individual differences in DPDR.

## 2 Study 1—Methods

### 2.1 Participants and Procedure

Study 1 used two datasets. First, data were collected from individuals with self‐reported DPDR. Originally, they were 275 individuals contacted between May 2023 and January 2024 through *Unreal* (unrealuk.org), a UK charity that raises awareness and promotes information on DPDR, as well as networking and sharing of DPDR lived experiences. The study was advertised via the charity's email newsletters, social media posts, and other media channels. Individuals were invited to take part in the study on a voluntary basis, with no incentives being offered. Participants were screened through the following prompt, informed by the definition provided by the United Kingdom's National Health Service (NHS) (2023) “Have you ever experienced either derealization and/or depersonalization symptoms? (Yes/No). As per the definition provided by the NHS: Depersonalization is where you have the feeling of being outside yourself and observing your actions, feelings or thoughts from a distance. Derealization is where you feel the world around is unreal. People and things around you may seem ‘lifeless’ or ‘foggy’. You can have depersonalization or derealization, or both together. It may last only a few moments or come and go over many years.”

In total, 160 individuals with self‐reported DPDR were included in the study and completed the procedure. The sample included 96 females (60.00%; *M*_age_ = 33.30, *SD*_age_ = 14.00), 48 males (30.00%; *M*_age_ = 35.40, *SD*_age_ = 14.80), and 16 who preferred not to report their gender (10.00%; *M*_age_ = 24.7, *SD*_age_ = 5.59). In addition, data from 303 individuals from the UK general population were used. They were recruited via Prolific (prolific.com) in June 2022. The sample included 149 females (49.17%; *M*_age_ = 40.30, *SD*_age_ = 13.00), 153 males (50.50%; *M*_age_ = 39.30, *SD*_age_ = 13.70), and one who preferred not to report their gender (0.33%; age = 26). Participants from the general population were invited to join a separate study and were compensated for their participation and time at the average rate of £9.72/h. The study procedure was the same between groups, consisting of filling out a set of online self‐report measures (about 15–20 min), through Qualtrics (qualtrics.com). The procedures obtained a favorable ethical opinion from the institutional ethics committee of the first, second, third, and fifth authors of the current manuscript.

### 2.2 Materials and Measures

Participants with self‐reported DPDR were administered the Cambridge Depersonalization Scale (CDS; Sierra and Berrios 2000). This is a 29‐item measure of the frequency and duration of DPDR symptoms over the last 6 months. For each item (e.g., “I have the feeling of being outside my body”), frequency and duration were assessed, respectively, using a 5‐point scale (*never/all of the time*) and a 6‐point scale (*few seconds/more than a week*). This scoring method is reported across a range of studies (e.g., Farrelly et al. 2016; Levin, Gornish, and Quigley 2022; Millman et al. 2022). However, the original version by Sierra and Berrios (2000) aggregated responses into a global score with frequency ranging between 0 (*never*) and 6 (*more than a week*) and duration between 1 (*few seconds*) and 4 (*about a day*). For this reason, a post‐hoc procedure was used to compare scores against the clinical cutoff indicated by Sierra and Berrios (2000), reported in the following paragraphs.

The 60‐Item Representation of the NEO PI‐R (NEO PI‐R‐60; Maples‐Keller et al. 2019) is a personality inventory based on the FFM (neuroticism, extraversion, openness to experience, agreeableness, conscientiousness). Items (e.g., “I am one who worries about things”) are rated on a scale from 1 (*very inaccurate*) to 5 (*very accurate*).

The Personality Inventory for DSM‐5—short form (PID‐5‐SF; Krueger et al. 2012) is a 25‐item version of the maladaptive personality inventory for the DSM‐5, measuring five traits of antagonism, detachment, disinhibition, negative affect, and psychoticism. Items (e.g., “I feel like I act totally on impulse”) are rated on a scale from 0 (*very false or often false*) to 3 (*very true or often true*).

Four distinct forms of perceived interpersonal support were measured through four items from Haslam et al.'s (2005) Social Support Scale, respectively, assessing the extent to which individuals feel that they receive the emotional support, help, advice, and material resources they need from the groups they are members of. Items were rated on a scale from 1 (*strongly disagree*) to 7 (*strongly agree*).

Finally, the UCLA 3‐item Loneliness Scale (UCLA‐3; Haslam et al. 2008) was used as an aggregated general measure of individuals' subjective feelings of loneliness. This is a measure of the extent to which individuals tend to feel, in their lives, a lack of companionship, to be left out, and isolated from others, respectively. Items are rated on a scale ranging from 1 (*hardly ever*) to 7 (*very often*).

### 2.3 Analytic Approach

Parametric independent sample's tests of mean differences, Cohen's *d* for effect sizes (interpreted as follows: small = 0.20–0.49; medium = 0.50–0.79; large ≥ 0.8), and false discovery rate (FDR) adjustments of *p*‐values were used. Multiple‐group graphical modeling was used to investigate and compare the topology, centrality, and interconnectivity of dispositional traits' networks in *individuals with DPDR* (henceforth: “DPDR”) versus *individuals from the general population* (‘Controls’). This used Spearman's correlations and EBICglasso regularization (Costantini et al. 2015; Epskamp and Fried 2018), following the testing of multivariate normality through the *b*_*1p*_ multivariate skewness coefficient (25.59, *p* < 0.001). The organization of the nodes into latent communities was detected by means of Louvain's algorithm (Blondel et al. 2008) and evaluated through modularity (*Q*), a measure of the strength of the division of the network into a set of subgraphs or communities, with values comprised between 0.3 and 0.7 indicating acceptable results (Epskamp, Borsboom, and Fried 2018). The centrality of nodes was assessed by means of expected influence (EI), a measure of *relative* centrality that accounts for enhancing and mitigating effects derived from signed connectivity between nodes. The graphical model was bootstrapped to allow for an evaluation of the stability of EI, obtained by implementing a case‐drop approach, whereby a varying portion of the original sample (i.e., from 5% to 75%, in steps of 5%, and 1000 samples examined at each stage), was progressively dropped, and the stability of the bootstrapped centrality was assessed using Correlation Stability (CS), that is, a measure of the highest fraction of cases that can be dropped whilst keeping a satisfactory correlation with the original sample (Epskamp and Fried 2018). Network predictability was also estimated and evaluated, indicating the degree to which a given node can be predicted by its neighbors.

For network comparisons, a three‐fold approach was used: (i) each network was estimated separately and visually inspected, then their characteristics were evaluated and compared; (ii) *Fused Joint Graphical Lasso* (Danaher, Wang, and Witten 2014) was used to model the data through contemporaneous multiple‐group network estimation and regularization, a powerful method that allows for the exploitation of similarities between graphical models whilst maximizing the information on their differences (Costantini and Epskamp 2017); (iii) a series of permutation tests (5000 iterations) investigated multiple‐group invariance in network structure, global centrality, and edges (van Borkulo et al. 2022).

The analyses were performed in R, version 4.3.1 (R Core Team, 2023), platform: x86_64‐pc‐linux‐gnu (64‐bit), using the following packages: *bootnet* (Epskamp, Borsboom, and Fried 2018), *EstimateGroupNetwork* (Costantini and Epskamp 2017), *igraph* (Csárdi et al. 2023), *mgm* (Haslbeck and Waldorp 2020), *NetworkComparisonTest* (van Borkulo et al. 2022), and *qgraph* (Epskamp, Borsboom, and Fried 2018).

### 2.4 Pre‐Registration, Data Availability, and Supplementary Material

The research was not preregistered. Data and code for studies 1 and 2 are publicly available to allow for full reproducibility of the results, at the following address: https://doi.org/10.17605/OSF.IO/5NXUY. In the interest of conciseness, all tables summarizing the demographic characteristics of participants (Table S1), descriptive statistics (Table S2), and correlation matrices (Table S3) are provided as Supporting Information. To protect the identity of participants, considering their vulnerability and sensitive topics, data from the semi‐structured interviews conducted in Study 3 are not shared.

### 2.5 Results and Discussion

Multivariate outliers' detection through Mahalanobis distances (*α* = 0.001) indicated no outliers. Data were validated post hoc, by considering intra‐individual variability (IVR) across all items' responses, using the procedure illustrated by Marjanovic et al. (2015). This consists of generating a random dataset of equal size to the empirical dataset and calculating IVR (i.e., standard deviation) across all observations. Then, logistic regression is run, using IVR as a predictor and the grouping variable (random vs. empirical) as a criterion. Classification accuracy (ratio of sensitivity and specificity; threshold = 0.50) > 0.80 indicates acceptable outcomes. In the current sample, classification accuracy was 0.90. In addition, no participants showed an IVR < 0.30.

Importantly, participants had self‐reported their experience of DPDR. This was assessed through a simple prompt and question, as mentioned in previous paragraphs. No formal screening or assessment was used to differentiate those with transient versus non‐transient forms or to identify participants based on the severity of their symptoms. For this reason, a post hoc procedure was implemented for rescaling CDS scores and comparing them against the clinical cutoff of > 70 (Sierra and Berrios 2000). This consisted of the following steps: (i) linear transformations of individual items' scores, so as to reflect Sierra and Berrios' (2000) original bounds (i.e., 0–6 for frequency items, 1–4 for duration items); (ii) computation of a global CDS score by summing up all individual items' scores; and (iii) comparison of the global CDS score against the established clinical cutoff. The procedure led to identifying 152/160 (95%) participants scoring > 70 (*M* = 161.00, *SD* = 45.40), suggesting probable clinical DPDR, while the mean score across the remaining eight participants (5%) was 62.10 (*SD* = 7.39).

Independent‐sample tests showed DPDR/Controls mean differences in conscientiousness (*t*_(461)_ = −3.83, *p*_FDR_ < 0.001, *d* = −0.37), neuroticism (*t*_(391.80)_ = 8.77, *p*_FDR_ < 0.001, *d* = 0.83), openness to experience (*t*_(461)_ = 2.84, *p*_FDR_ = 0.007, *d* = 0.28), loneliness (*t*_(461)_ = 6.40, *p*_FDR_ < 0.001, *d* = 0.63), antagonism (*t*_(461)_ = 3.18, *p*_FDR_ = 0.003, *d* = 0.31), detachment (*t*_(461)_ = 6.26, *p*_FDR_ < 0.001, *d* = 0.61), disinhibition (*t*_(461)_ = 3.10, *p*_FDR_ = 0.003, *d* = 0.30), negative affect (*t*_(394.74)_ = 9.72, *p*_FDR_ < 0.001, *d* = 0.91), psychoticism (*t*_(461)_ = 14.58, *p*_FDR_ < 0.001, *d* = 1.42), and support (*t*_(374.38)_ = −2.81, *p*_FDR_ < 0.001, *d* = −0.27). A detailed visual representation of the results is available as Figure S1.

Results from separate graphical models showed moderate and positive associations between psychoticism and negative affect, between psychoticism and disinhibition, and a negative association between psychoticism and conscientiousness. However, in Controls, but not in DPDR, psychoticism was positively associated with detachment and antagonism. Openness was negatively associated with detachment in both groups, whereas in Controls, openness was also positively associated with extraversion, agreeableness, and psychoticism. Perceived support was negatively associated with detachment and loneliness in both groups; only in Controls, perceived support was positively associated with extraversion and agreeableness.

**FIGURE 1:**
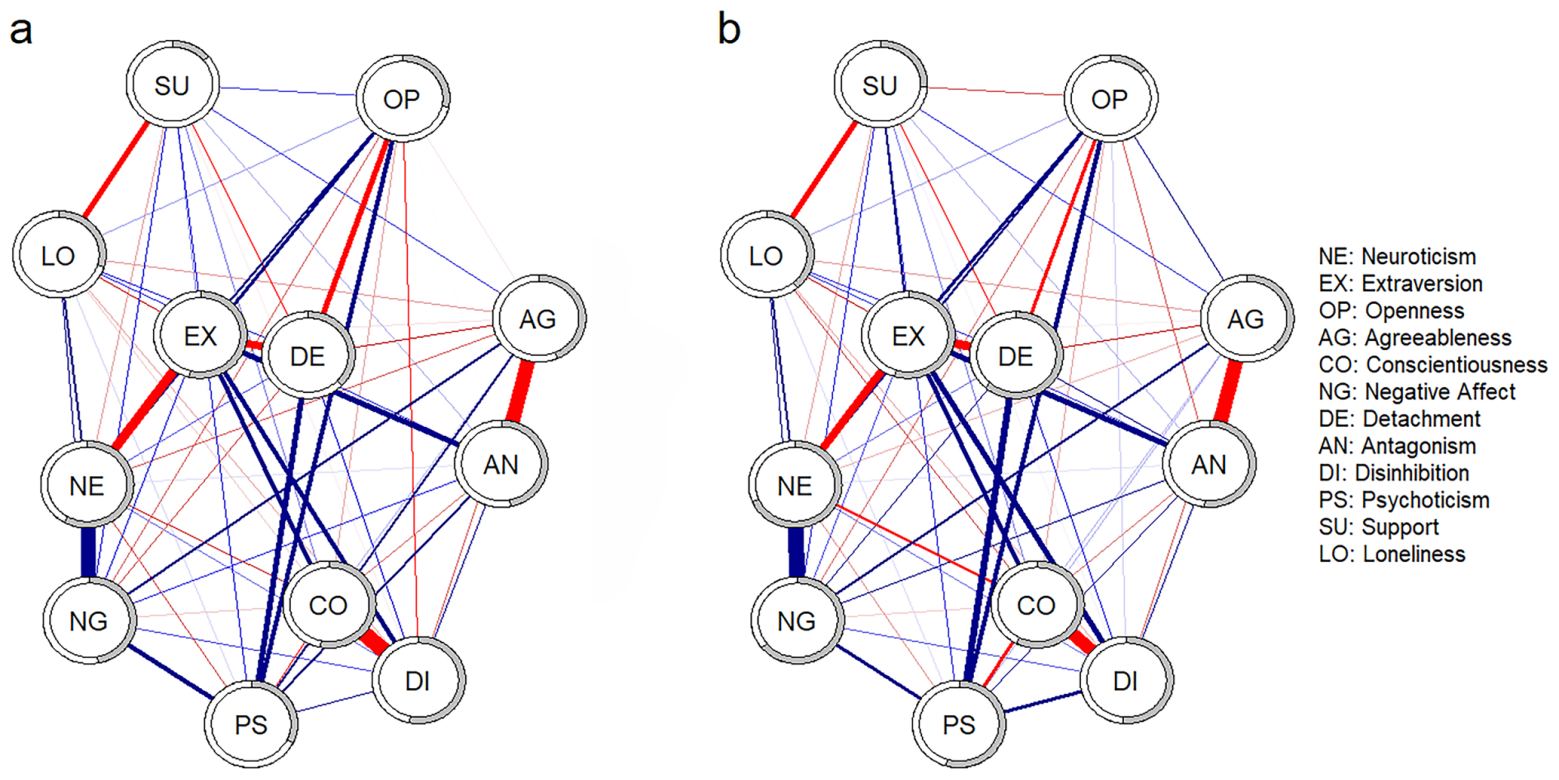
Study 1, Fused Joint Graphical Lasso graphical models: (a) individuals with self‐reported DPDR (*n* = 160); (b) individuals from the general population (*n* = 303). Red edges indicate negative associations.

Both networks showed satisfactory and approximately comparable levels of average predictability (DPDR = 0.41 vs. Controls = 0.48) and distributions between groups, with neuroticism (0.58 and 0.68) and extraversion (0.54 and 0.55) being the most predictable nodes and perceived support (0.14 and 0.26) and loneliness (0.29 and 0.39) among the least predictable ones. Community detection was acceptable for DPDR but not Controls (*Q*_DPDR_ = 0.42 vs. *Q*_Controls_ = 0.23), and for this reason, it was decided to avoid comparisons related to latent clustering. Centrality estimates were stable in Controls but less so in DPDR (CS_EI,DPDR_ = 0.30; CS_EI,Controls_ = 0.70). Regarding EI estimates from the joint model, the highest values were for negative affect (2.00 vs. 2.13), psychoticism (1.64 vs. 1.33), and neuroticism (0.37 vs. 0.30), in both groups. However, loneliness was found to be lowly central in Controls (−0.50) and averagely central in DPDR (−0.18). Figure 1 represents the jointly estimated networks. Supporting Information 1 includes a detailed weight matrix (Table S4). Supporting Information 1 includes line plots of EI centrality estimates (Figure S2).

The visual inspection of the jointly estimated networks showed a greater level of association between psychoticism and agreeableness in DPDR versus Controls, whereas in Controls, the negative association between openness and disinhibition and the positive association between openness and perceived support observed in DPDR were inverted in sign. The link between negative affect and detachment was negative in DPDR and positive in Controls. However, no significant differences were found between DPDR and Controls in network structure (*M* = 0.25, *p* = 0.07) or edges, but a significant difference was found in global EI (*S* = 1.61, *p* = 0.03; EI_DPDR_ = −0.86, EI_Controls_ = 0.76) (see Supporting Information 1, Tables S5 and S6).

These results overall indicate no substantial differences in the way personality dispositions are conditionally associated in a sample of individuals with DPDR versus a general population sample. However, large mean differences were observed in psychoticism and negative affect, followed by FFM neuroticism, perceptions of loneliness, and PID‐5 detachment. The effects observed for support, antagonism, disinhibition, openness to experience, and conscientiousness were significant but comparatively small. Overall, these findings prompted further investigation of the conditional associations between DPDR traits, personality, perceived support, and loneliness in individuals with DPDR.

## 3 Study 2—Methods

### 3.1 Participants and Procedure

Study 2 used the same dataset from Study 1, including a sample of 160 individuals with self‐reported DPDR (see previous paragraphs for a detailed description).

### 3.2 Materials and Measures

Study 2 used the same measures of personality, perceived social support and loneliness, and DPDR as Study 1. Additionally, the analysis used two separate DPDR scores, obtained by averaging CDS frequency and CDS duration scores, respectively, to model multivariate conditional dependencies between these important aspects of DPDR, personality, and perceptions of support and loneliness.

### 3.3 Analytic Approach

Study 2 used a graphical modeling approach and the same estimation methods used in Study 1. For centrality, in addition to EI, Bridge EI (BEI) was also used and the relevant CS computed. BEI is a measure indicating the sum of the edges extending from a given node and the indirect effect of a node on external communities through other nodes (Jones, Ma, and McNally 2021).

### 3.4 Results and Discussion

**FIGURE 2:**
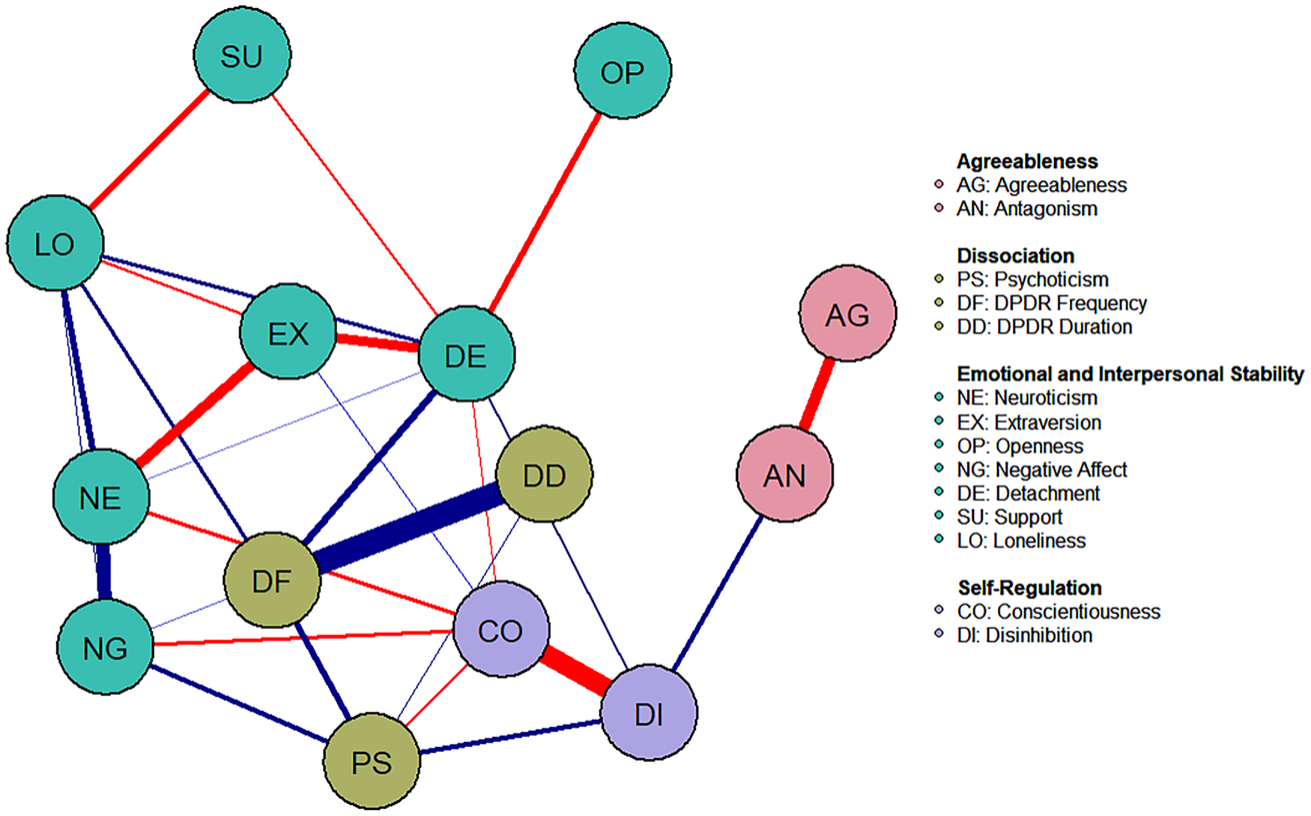
Study 2, Graphical model with Spearman's correlations and EBICglasso regularization (*N*_DPDR_ = 160).

The results from network analysis showed that DPDR frequency and duration were positively and highly associated, as expected. DPDR frequency was also moderately and positively associated with psychoticism, detachment, subjective feelings of loneliness, and negative affect. DPDR duration was only positively associated with psychoticism, besides its relationship with DPDR frequency. Psychoticism was, in turn, negatively associated with conscientiousness and positively with disinhibition and with negative affect. Figure 2 represents the network model.

Four latent communities were identified as best candidates to represent the graphical model (*Q* = 0.47), renamed and summarized as follows: (i) *Agreeableness*, identified by agreeableness and antagonism; (ii) *Self‐regulation*, including conscientiousness and disinhibition; (iii) *Dissociation*, including DPDR frequency and duration and psychoticism; (iv) *Emotional and Interpersonal Stability*, including neuroticism, extraversion, openness to experience, negative affect, detachment, perceived support, and loneliness. Antagonism (EI = −0.12, BEI = 0.05), disinhibition (EI = −0.23, BEI = 0.01), negative affect (EI = 0.24, BEI = 0.02), and DPDR frequency (EI = 0.63, BEI = 0.11) were the most central nodes within their relevant communities, although detachment (EI = −0.07, BEI = 0.09) and psychoticism (EI = 0.16, BEI = 0.08) showed the highest BEI, indicating their potential mediating role between communities. However, EI (CS = 0.65) but not BEI (CS = 0.15) showed satisfactory stability, requiring caution in the interpretation of BEI. The network showed acceptable predictability across all nodes (46%). Supporting Information 1 includes line plots of centrality estimates (Figure S3).

These findings suggest a role for psychoticism in DPDR, namely, a tendency to experience egodystonic inner/outer states that may prevent individuals with DPDR from adequately regulating their inner perceptions with environmental and contextual factors (Piedmont et al. 2009). Psychoticism may predispose individuals to a chaotic organization of their subjective experience, an inability to codify and appraise different types of stimuli, and a cycle of emotional instability, anxiety, and rumination, commonly observed in individuals with DPDR symptoms (Medford et al. 2005). This further helps explain the anxiety and sense of impairment that these individuals often report. On a related note, the analysis showed that detachment was external to the community of dissociative traits, challenging the idea that DP and DR cluster with generic tendencies to withdrawal or detachment from internal/external stimuli, rather, they may reflect specific dispositions to a dysregulated perception and impaired appraisal of them (Ciaunica et al. 2022; Medford et al. 2005; Soffer‐Dudek 2023).

## 4 Study 3—Methods

### 4.1 Participants and Procedure

Study 3 was based on data from an opportunity sample of adult individuals with self‐reported DPDR. Similarly to studies 1 and 2, participants were recruited in collaboration with *Unreal*. To be eligible to participate, individuals had to be ≥ 18 years and have a self‐reported history of DPDR. The same screening procedure used in Study 2 was used in Study 3. Participation was voluntary and required the following, in order: (i) read and understand a detailed information sheet: (ii) sign an electronic consent form and complete a short online survey via Qualtrics; (iii) attend a ~60‐min online interview via Microsoft Teams. Interviews were conducted by the principal investigator (first author of the present manuscript) and a research assistant (second author) and were based on a semi‐structured protocol that required participants to engage with storytelling on targeted aspects of their lived experience of DPDR. Data saturation was achieved when the researchers considered the quantity and quality of the information to be adequate to answer the research questions and make any further data and coding redundant (Fusch and Ness 2015).

When taking the online survey, participants were asked to answer a set of demographic and other questions, including the CDS. They were invited to consent to establish a link between their survey data and interview transcripts by means of a unique identifier of their choosing. Nineteen individuals completed the procedure (*n* = 13 UK residents, and *n* = 6 residents in other countries). The sample included *n* = 6 females (31.58%; *M*_age_ = 33.20, *SD*_age_ = 13.60), *n* = 4 males (21.05%; *M*_age_ = 35.80, *SD*_age_ = 14.20), and *n* = 4 individuals who preferred not to report their gender (21.05%; *M*_age_ = 24.90, *SD*_age_ = 5.43). Some participants (*n* = 5, 26.31%) could not retrieve their unique identifier at the time of the interview, making their demographics and CDS data unknown. Post hoc analysis showed that all participants who reported their identifier at the time of the interview, and for whom CDS data were available, scored > 70 at the CDS, indicating probable clinical DPDR. The procedure was reviewed and obtained favorable opinion from the institutional ethics committee of the second, third, and fifth authors. Supporting Information 1 include additional demographic information of participants (Table S7).

### 4.2 Analytic Approach and Materials

Study 3 used a qualitative methodology to explore participants' characteristic adaptations to DPDR and narrative identity reconstructions, aiming to map their journey through making sense of, adapting to, and coping with DPDR, answering the questions as to whether/how DPDR affects their sense of who they are, the beliefs in their own capacity to influence the course of their lives, and their relationships with others. This methodology is informed by the theoretical framework of the *Life Story Model of Identity* (LSMI) (McAdams 1988, 2018) and *Complex Adaptive System* (CAS; Kerr 2019). The LSMI and CAS posit nonlinear dynamical trajectories of individual development, highlighting the unique, complex, and multifaceted nature of one's characteristic adaptations to life events and identity reconstructions. Characteristic adaptations and identity reconstructions articulate across distinctive topical themes that underlie individuals' narratives, namely: *redemption* (i.e., negative/positive life transitions), *contamination* (the opposite of redemption), *agency* (perceived autonomy and influence) *communion* (motivation to form and maintain interpersonal/intimate relationships and group affiliations, including the sense of belonging and identification that individuals derive from them), *self‐exploration* (reflecting upon oneself through reconstructing and narrating own stories), *coherent resolution* (coherent narrative closure/ending), and *meaning‐making* (integrating narrative themes into subjective meaning, purpose, and prospective action) (Kerr 2019; McAdams and McLean 2013).

Specifically, the study's analytic approach consisted of a mixed inductive/deductive thematic analysis (see Braun and Clarke 2021), which allowed for a comprehensive and flexible exploration of the themes underlying participants' narratives while using the LMSI/CAS framework as an aid in coding and interpretation. For this purpose, an ad‐hoc semi‐structured interview protocol was designed and used to collect data, which included a series of pre‐established prompts of autobiographical recollections of participants' experience of DPDR (e.g., onset, development, management of symptoms), characteristic adaptations (e.g., attributional beliefs and coping strategies), and narrative identity reconstructions (e.g., whether/how DPDR affected their sense of who they are, their agency and communion).

Data generation, thematic analysis, and interpretation involved the following steps, in order: (i) online interviews, conducted and recorded online, via Microsoft Teams, with participants clearly being informed about the recording and requested to provide their consent ahead of the scheduled appointment. The interviews were automatically transcribed via *Microsoft's Transcription* and *Closed Captions* functions; (ii) the second author of the manuscript manually reviewed the transcriptions across two rounds of revisions, checking the completeness and accuracy of the text against the content of the audio files and ensuring that any identifiable information was omitted and pseudonymized, when required; (iii) the first and second authors of the manuscript familiarized themselves with the data by engaging in multiple readings of the interview transcripts, then, they iteratively coded narratives into themes across a series of both individual and group sessions, reviewed the coding as the analysis progressed, and discussed the findings until consensus was achieved about the redundancy of any further coding and interpretations; (iv) the first and second authors drafted and completed the report of findings, which was eventually reviewed and approved by all authors.

### 4.3 Results and Discussion

Four main themes were identified, reflecting participants' narrative identity reconstructions, summarized as follows: (i) “How it started and what happened next”: DPDR onset as a major life turning point and contaminator; (ii) “Why me?” Meaning‐making, exploratory narratives associated with the lived experience of DPDR; (iii) “The more you think about it, the worse it gets”: Coping strategies, tentative redemptions, and failed resolutions. In the following paragraphs, these themes are explored and evaluated in‐depth, supported by a selection of participants' quotes. Supporting Information 1 include a summary table of themes (Table S8).

#### 4.3.1 Theme 1: “How It Started and What Happened Next”: DPDR Onset as a Major Life Turning Point and Contaminator

Most participants dated back the onset of DPDR symptoms to either childhood or teenage years, describing the condition in terms of a contamination scene, that is, major negative turning points in their lives. Narratives were commonly characterized by feelings of surprise and unprecedented mental confusion associated with the appearance of highly disruptive alterations to their perception of themselves and their surroundings.

I've been saying since I was tiny, tiny to my parents, things like I don't feel like I'm here. I don't feel like connected to myself. I don't recognize the person in the mirror. (Laura)

DPDR was immediately perceived as intense, odd, estranging, ultimately challenging participants' ability to make sense of newly blurred perceptual boundaries. Interestingly, some participants identified derealization as chronologically antecedent to depersonalization. In no cases, participants reported failures in reality testing. Most participants early achieved the realization that symptoms were stable, combined with the emotional shock and sometimes even terror of falling onto a characteristic cycle of anxiety and rumination associated with them.

Yes. So, when my symptoms were the worst, I was obsessing, I thought I had schizophrenia. And I was obsessing about that. And I think it was getting worse. Even these days, I'm always kind of obsessing about self‐help and trying to get better. Like, every second, I have ruminations about how to fix myself, this kind of thing. (Jack)

That will then have ripple effects throughout the rest of your life. So, I went to university, but it was really difficult because these symptoms are getting worse. (Toby)

This experience was commonly accompanied by an inability to describe, characterize, or simply communicate the nature of symptoms, even at a basic level. In fact, most participants reported major difficulties in interpreting symptoms, to the point that attempting to describe them to others was considered challenging and potentially counterproductive, often relying on the belief that nobody could understand their struggle, especially around the time when symptoms first occurred.

All I could say was I don't feel real. You know, that was about the best way I could describe it, actually. I was in a hospital undergoing neurological tests to try and figure out what was going on, and I'm, like, “I'm not crazy, you know. I'm feeling… I'm experiencing something real that is unreal at the same time.” And it was basically my world becoming two dimensional. At least that's the best way I could describe it. (Dan)

For some of them, this experience prompted greater existential concerns about themselves and the world, a theme that is well‐known in literature (Ciaunica et al. 2023). Moreover, in most cases, the incommunicability of symptoms did not seem to improve or resolve through access to primary or specialized care, which in turn, triggered feelings of isolation and hopelessness.

I'd go and speak to medical professionals, and no one would have a clue what it was. It was everything from being completely dismissed and the doctor gets up and shows you the door and goes “it's probably nothing, get out.” But I need help and it's just like “you don't get it, do you”? Complete lack of awareness in medical communities, which when you're going through something you don't know what it is, and no one can tell you. That's ten times worse than having a name for it, but the terror of not knowing what it was! (Toby)

#### 4.3.2 Theme 2: “Why Me?” Meaning‐Making, Exploratory Narratives Associated With the Lived Experience of DPDR

Participants' autobiographical explorations associated the onset and development of DPDR with a diverse range of life antecedents. Some narratives pointed out the use of recreational drugs, while others indicated traumatic events (e.g., family‐related, and emotional struggles, interpersonal conflict at home or school), or physical procedures (e.g., hospitalizations and surgery), whereas a group of them reported no recollection whatsoever as to what might have triggered DPDR. In fact, it was common not to comprehensively remember any developmental trauma, not even minor negative experiences, which sometimes made the appearance of symptoms even more disorienting, challenging individuals' ability to understand and make sense of what and why was happening to them.

Trying to see what can be happening in my life that makes me feel like this way or something, but I cannot, really. I haven't grasped something really particular that I can think of; that would have been of great help to just kind of make these symptoms just disappear or fade or something. (James)

All participants emphasized the intensity and discomfort associated with derealization and the subsequent feeling of alarm of losing control or “getting crazy.” Incredulity, dismay, but at the same time, fixation on symptoms often led to incessant questioning over the cohesiveness of one's own self and identity, as if the hypothesis that they are actual perceivers and in control of their own perceptual processes had to be continuously tested (e.g., see Ciaunica et al. 2022). This was often accompanied by the fear, in social interactions, that others may find out about this state of absorption and rumination.

So, when I have a deep… when I feel that loss of identity, it's very hard to connect with others, and I can…. I remember… I can, like, I don't know what to say to people and I feel very… yeah, it's not natural anymore. So, I feel very disconnected, and I feel like I'm just pretending to have social interactions […]. I don't know if this is caused by my symptoms, but I feel detached, and you share less things with others. And maybe I don't display a lot of emotions. And this has been a kind of a problem in some relationships. (Jack)

#### 4.3.3 Theme 3: “The More You Think About It, the Worse It Gets”: Coping Strategies, Tentative Redemptions, and Failed Resolutions

Characteristic adaptations found in the study can be divided into two main sub‐themes, respectively related to the management of short‐term feelings of anxiety, rumination, and fear, especially during acute episodes, and long‐term coping strategies and approaches to recovery. Regarding the former, a common sense‐based approach that reportedly helped relieve symptoms was *distraction*. This involves engaging in mundane tasks, everyday activities, and conversations, which several participants reported as helping them at least temporarily re‐focus their mind upon external/contextual elements versus their altered inner experiences, achieving some form of intero‐exteroceptive integration that they experienced as “normal,” ultimately carrying a knock‐on positive effect on their well‐being.

So, usually, when I'm not being introspective, when I'm not thinking in my head and when I force myself to be to think outwardly and to focus on outward things and kind of preoccupy my mind. That's when my mind gets the mental relief that it needs to decompress and to recover. So, if I do something that stimulates my mind outwardly, like an action, rather than a thought, then that helps me. So, if I get up from where I'm sitting and I tidy up, or if I get up and I walk outside, or if I do something that is a really interest of mine or that actually captures my attention – which is hard when you've got DPDR – such as arts, or cooking, or something creative and expressive, that helps me process emotions and give my mind a break as well. Helps the symptoms kind of, you know, alleviate a little bit. (Rose)

Some even explicitly argued that coping with DPDR “does not work like that,” referring to the potential risk of continuously paying attention to and of talking about the problem to act as a trigger and reinforce the frequency and duration of symptoms, versus the benefits of accepting the condition, almost as if losing interest in the incessant and ruminative nature of symptoms and carrying on with life helped sustain their well‐being.

It's something that I live with. It's like a chronic condition. As I'm getting older, I'm experiencing all sorts of physical things as a result of being older and at the time, you know, I hurt a part of my body running. At the time, I thought, I cannot live like this. I cannot live with the constant pain. Five years down the line, it still hurts, but I deal with it. It just becomes your new normal. So, that's exactly how I treat the depersonalization, that it's just my baseline state. And if I think about it, it stresses me out. So, I don't think about it. That is the general way that I cope. (Liz)

As for long‐term coping and approaches to recovery, narratives highlighted diverse and not always coherent reports of experiences. A common positive turning point in narratives coincided with hearing or learning about symptoms called “derealization” or “depersonalization,” to the point that most of participants could all exactly remember when and how that happened (most often, through the Internet), and the feelings of validation henceforth associated with it.

I ended up, I think, when I was… honestly… 15, I found – it was like a forum or a chat online, quite an obscure one – but it was people talking about DPDR, and all of the things they were saying were basically describing my situation exactly. So, I ended up sort of talking with them, and they were, like: “yeah, that sounds exactly like what I'm going through.” So that was very much like a turning point of, okay, like, you're not crazy. It's genuinely something people know about and can quantify, and you can understand it. (Ola)

Narratives commonly highlighted that knowing about DPDR often was associated to a subsequent failed redemption, that is, an unmet promise of recovery by circumstances and actors that did not help.

I haven't ever been diagnosed. I've been trying to for the longest time, but I feel like I've been sort of misunderstood, like I go to my GP [General Practitioner] about it, and they just refer me to somewhere else and they don't really know what to do with me. Like, I've been through, I think, every therapy service in […] that I can for free, and they've said “You're not making any improvements. Your questionnaire results are exactly the same as when we started this, three months ago.” I don't think they're really equipped to understand or deal with these sort of symptoms, and no one really seems to know what to do about it. (Lara)

Regarding treatments, eye‐movement desensitization therapy, cognitive‐behavioral counseling and forms of psychotherapy seemed to work for some, but frequently, reportedly to a limited extent. However, participants' stories mostly often indicated experiences with healthcare and mental health professionals who were perceived as unprepared to recognize, diagnose, and treat DPDR, sometimes reportedly recommending ineffective remedies, which ended up reinforcing feelings of hopelessness, loneliness, and isolation.

#### 4.3.4 Theme 4: “It's Kind of Hindered Things for Me”: DPDR, Disrupted Agency, and Communion

Participants articulated agency and communion as inherently intertwined and mutually reinforcing themes in their experience of DPDR. In this regard, their stories associated the DPDR‐related perception of losing control with the belief of not being able to function interpersonally or even experiencing any form of affection towards others. Metaphors such as feeling like living “in a dream” or “like a robot” were common and associated with poor general and interpersonal self‐efficacy beliefs. Often, these beliefs seemed to consolidate into negative self‐esteem and a diminished sense of self and identity, which in turn, prompted self‐descriptions and categorizations in terms of a mentally “impaired” or “broken” person, underlying feelings of shame and internalized stigma, ultimately worsening the experience of DPDR.

If I am feeling really spaced out, or really, like really derealized, really depersonalized. I think, because it feels so, I feel so disconnected from myself. I just struggle to do anything that revolves around my personality. So, if I'm meeting a new person, then I suddenly get a sort of, don't know, I feel like it comes in waves, almost. If I get like a wave of derealization or something like that I feel like I'm speaking on behalf of someone else, because there's a disconnect between me speaking and me because of the derealization. So, it's like I can't introduce myself properly, ‘cause I feel like I'm… I'm talking on behalf of someone else. Yeah, so that could usually end up with me embarrassingly explaining why this is happening. (Ola)

Forms of negative identifications are described in the literature in terms of a “curse” (Schury, Nater, and Häusser 2020): Individuals naturally aspire to pursue and preserve a positive, continuous, and distinctive self, which in turn, revolves around the perceptions that are most salient in one's own experience (Ryan and Deci 2001). Accordingly, most participants in the present study reported difficulties in establishing a sense of self and identity that goes beyond the pervasive experience of being someone who lives with DPDR, which in turn, affected their view of themselves as effective agents over a range of life domains (e.g., social networking, education, work) and prevented them from setting and pursuing a vast number of life goals. For example, some reported that living with DPDR had impaired their ability to cope with everyday tasks and maintain stable interpersonal relationships, pursue group memberships, affiliations, and career goals.

I tend to steer away from most people because, uhm, with DPDR It's really difficult to have a conversation. Sometimes, I get frozen with anxiety. I don't know what to say in conversations, and people tend to dismiss me quite easily. So, yeah, I've struggled a lot. It's stopped me from trusting people in relationships because I can't feel present in the moment. I believe it's stopped me from having a husband and kids. Yeah. And it's also stopping me from being a teacher, which is what I really want to do, as well. (Rose)

This might also help explain why some participants considered talking about DPDR as dysfunctional, often triggering a sort of “contagion” and reinforcement of the symptomology, that is, what previous literature described in terms of a transmission of a mental response from a distressed individual to an observer via verbal and non‐verbal interaction (Schury, Nater, and Häusser 2020).

Don't want to be around anybody has a mental illness. That's a no. It just brings me down. But you know, cause I don't want to be sitting around this: “let's talk about our mental illness.” No, that's not what I want. When you talk about it, it's there, it's part of mine. That's not what. I want, it's not what runs my life. (Dan)

Nevertheless, when available, support offered by close ones (e.g., family, romantic partners, friends) seemed to mitigate the negative impact of identifying as someone living with the condition by reducing the perception of disruptiveness of DPDR symptoms and enhancing individuals' agency and well‐being. Forms of effective support were reportedly characterized by others' awareness of participants' struggles with DPDR and a contemporaneous empathetic, non‐judgmental, and lenient acceptance of it, which often served the basis for reinstating agency and help participants find forms of meaning and purpose in life.

Oh, yeah, and it really helps me. Yeah. Really helps me to share. So very few people know that I feel this, but for example, my boyfriend really understands. I think he really is a very big support. So, even on a daily basis, I like to tell him, like, today I'm feeling a little bit more decent, and It's reassuring to know that, OK, he knows that; he has this information and maybe he will also try to help me. So, it really helps to share. (Maria)

## 5 General Discussion

The aim of the present research was to explore individual differences in DPDR by means of three studies, respectively (i) comparing mean differences, topology, and interconnectivity of dispositional broadband traits (FFM), perceived social support and loneliness, and maladaptive trait domains (PID‐5) in individuals with DPDR versus individuals from the general population; (ii) evaluate a network of broadband/maladaptive traits, perceptions of social support and loneliness, and DPDR traits (frequency/duration) in individuals with self‐reported DPDR; and (iii) explore characteristic adaptations to DPDR and narrative identities of people with lived experience of the condition. The results of Study 1 showed significant mean differences across all variables, with greater effect sizes observed for psychoticism and negative affect, followed by neuroticism, loneliness, and detachment. The visual inspection of the jointly estimated networks showed a greater level of association between psychoticism and agreeableness in individuals with DPDR versus Controls from the general population. Negative affect and detachment were negatively correlated in DPDR and positively in Controls. However, significant differences were not found between network structures or edges, but in the global centrality of nodes, that is, the total level of nodes' interconnectivity. Study 2 expanded the results from Study 1 by re‐analyzing the data from the DPDR sample in a way to prioritize the study of the relationships between DPDR traits (frequency and duration of symptoms) and the organization of the network in latent clusters or communities. The results showed a community of dissociative tendencies formed by interrelations between DPDR traits and psychoticism. Another latent community of emotional stability was connected to DPDR through detachment, whereas conscientiousness and disinhibition formed a third community of self‐regulation connected with DPDR traits through psychoticism. Lastly, Study 3 investigated characteristic adaptations and narrative identity reconstructions by exploring and interpreting the main themes underlying stories of DPDR lived experiences. Expectedly, the onset and development of DPDR was commonly indicated as a major episode of contamination, that is, a key life transition from a condition of well‐being to a pathological state, with narratives pointing out incommunicability, and subsequently, disrupted agency and isolation as crucial themes to understand the struggle with building up a coherent, distinctive, and continuous sense of self for those affected by the condition. Misdiagnosis and ineffective treatments were reported as common and reinforcing the cycle of perceptual dysregulation/anxiety/rumination/isolation that characterizes DPDR, whereas distraction and self/other acceptance were associated with short‐ and long‐term benefits, including a greater sense of agency, better interpersonal relationships, enhanced social support, and self‐actualization.

To the best of the authors' knowledge, this is the first research that has investigated individual differences in DPDR using a theoretical framework that integrates trait theory with the LSMI (McAdams 1988, 2018) and CAS (Kerr 2019) perspectives. These results indicate individual differences in perceptual dysregulation as key in DPDR and shed light on the maladaptive organization of personality traits in those affected by the condition. The findings of the present research align with recent literature that points out perceptual dysregulation as an essential mechanism in the interpretation of DPDR symptoms (Ciaunica et al. 2022, 2023), summarized by the relationships between DPDR traits and PID‐5 psychoticism, which previous literature had already found in association with dissociative tendencies (Ashton et al. 2012). They highlight that although the structure and edges of the network of those living with DPDR may not significantly differ from counterparts from the general population, specific links between DPDR and maladaptive trait domains of personality exist. Regarding the non‐significant differences observed in network structures and edges, three possible explanations are offered, as follows: (i) measurement variance may play a role, warranting replication of results through alternative measures of broadband and maladaptive personality; (ii) individual differences in DPDR may be detected at the level of the location of individuals upon latent traits' continua, rather than in the relationships between traits; and (iii) despite the effort to recruit from a hard‐to‐reach population, the sample size may not ensure sensitivity to detect smaller effects. Nevertheless, the latent community formed by DPDR traits and psychoticism in the present study's sample highlights the characterization of DPDR in terms of patterns of odd thinking and dystonic self and world‐perceptions, confirming findings from recent research (Ciaunica et al. 2023; Piedmont et al. 2009). Dysregulated perception prompts a chaotic organization of the subjective experience, an inability to adequately codify and appraise different types of inner and outer stimuli, prompting a cycle of anxiety, obsessive rumination, disrupted self‐esteem and poor beliefs of self‐efficacy, and social isolation (Medford et al. 2005). Moreover, the findings from the present study showed that PID‐5 detachment was external to the community of dissociative traits, supporting the idea that DPDR may represent a form of hyperfocus on perceptual experiences, whereas withdrawal, anhedonia, and intimacy avoidance may be second‐hand products of the impact of DPDR on individuals rather than a primary characterization of the condition. Nevertheless, the results from Study 1 showed a negative association between openness and disinhibition in individuals with DPDR and a positive association between the latter and psychoticism. Higher levels of antagonism, disinhibition, and openness to experience, and lower levels of conscientiousness were also found in the DPDR group versus individuals from the general population. However, when considered in association with DPDR traits, in Study 2, the link between psychoticism and disinhibition disappeared, and the differences between edges in Study 1 were not significant, anyway. On the other hand, a consistent positive association between disinhibition and psychoticism was found in both studies, which may explain a tendency to unrestrained and unusual forms of thinking observed in DPDR. These results support previous literature that showed associations between experiential permeability (Piedmont et al. 2009), defined in terms of the “ability of an individual to regulate interactions between the inner world of experiences and the outer reality of activities and relationships” (p. 1247), and psychoticism, schizotypy, dissociative experiences, and also sleep problems, regardless of stress levels (Tan et al. 2018). In particular, these findings suggest that DPDR may be associated, on the one hand, with a tendency towards intellectual curiosity and perceptual stimulation, and on the other hand, with an orientation towards unrestrained forms of thinking. Consistently, Ashton et al. (2012) found that openness and psychoticism were lowly but significantly loaded onto a single latent factor of schizotypal and dissociative tendencies. High levels of psychoticism and openness to experience may therefore be implicated in individual differences in DPDR, possibly entailing a characteristic inability to set functional boundaries between inner and outer stimuli that end up challenging an individual's natural mastery of one's own thoughts and perceptions (Ciaunica et al. 2022). In addition, there is a known link between openness and overt rebelliousness, which may further explain the results of high antagonism and low consciousness found in the present study and corroborate the idea of overlaps between DPDR and experiential permeability (Piedmont et al. 2009).

Regarding characteristic adaptations and narrative identity reconstructions, participants described DPDR in terms of a major life contaminator, that is, a turning point that determined a critical transition from a positive to a negative life condition, in all cases, yet to be resolved. This aligns with theoretical perspectives considering individual difficulty in the integration of trauma experience and narratives where dissociation is experienced potentially leading to feelings of experiential contamination and exacerbating distress (McAdams et al. 2001). The difficulty in making sense of the new experience of blurred boundaries between oneself and one's surroundings was central in participants' reports of derealization. Although presenting some variations in scenes and characters, all participants shared stories of strain and distress in adapting to this experience, perceived as shocking and constantly threatening to their sense of self, which in turn, affected their ability to bond, pursue educational/work attainments, and construct a positive and functional social identity. Some coping strategies were mentioned and explored; however, most often, they proved ineffective, especially considering the difficulties experienced in communicating symptoms with others, including healthcare professionals.

Nevertheless, diversion, emotional closeness, and empathic acceptance by others provided some forms of relief and support to those with DPDR. Furthermore, most participants reported obsessive/intrusive thoughts and hyperfocus on their dysregulated perception as characterizing their experience of DPDR. The link between obsessive and compulsive forms of thinking and reinforcement of DPDR symptoms is known in the literature (Ciaunica et al. 2023; Medford et al. 2005; Soffer‐Dudek 2023; Torch 1978). However, their role in the onset and development of DPDR may have been long overlooked. Future research may benefit from investigating whether these tendencies correspond to a premorbid obsessive‐compulsive personality configuration rather than a comorbid obsessive‐compulsive disorder. This may help test hypotheses on outcomes of interventions targeting obsessive and ruminative thinking to help break the cycle of hyperfocus on estranged perceptions that characterize the experience of DPDR.

These results also suggest other potential pathways for assessment and intervention for DPDR. First, they indicate that PID‐5 psychoticism represents a useful target to integrate with specific measures of DPDR traits, an aid in clinical settings that may help improve the timing and accuracy of assessment and diagnosis. Second, they suggest that breaking the vicious cycle of perceptual exploration and rumination may also help achieving better therapeutical outcomes. Nevertheless, they indicate that counseling and psychotherapy should also target feelings of loneliness and social isolation that were found to be significantly higher in the DPDR sample versus individuals from the general population, perhaps by helping those affected by the condition find forms of social bonding that foster acceptance and emotional support.

In conclusion, the present research highlights individual differences in perceptual dysregulation as characterizing the experience of DPDR, as indicated by the large effect found in the PID‐5 trait domain of psychoticism and the organization of DPDR traits and psychoticism into a latent cluster, within the relevant network model. Moreover, they confirm that DPDR significantly affects individuals' ability to adapt to and cope with life challenges, with most participants reporting disrupted agency, communion, and a fragmented identity that hinders their pathway to well‐being and self‐actualization.

This research has limitations. First, in recruiting for studies 1 and 2, no standard assessment was used to screen participants. This would have strengthened the sampling strategy and helped differentiate individuals with stable symptomology from others with transient experiences, despite the probable clinical relevance of symptoms in almost the entirety of participants, as reported in the previous paragraphs. Second, no information about mental health/neurological history was collected. Third, the sampling strategy may have favored self‐selection of individuals with current forms of DPDR, and for this reason, future research comparing these findings with data generated from successful DPDR recovery stories may be of great interest and value. Fourth, the sample's size may have impacted the power to detect small effects in the data, and as such, replication in larger samples is warranted. Fifth, the study was cross‐sectional, limiting the type of inference being pursued. Lastly, the study did not consider a range of additional constructs that could inform the understanding of individual differences in DPDR, for example, experiential permeability and obsessive‐compulsive personality, which as discussed, have the potential to inform future research, assessment, and intervention.

## Author Contributions

**Emanuele Fino:** conceptualization, methodology, software, validation, formal analysis, investigation, resources, data curation, writing – original draft, writing – reviewing and editing, visualization, supervision, project administration. **Thalia Jemmett‐Skinner:** methodology, software, formal analysis, investigation, data curation, writing – original draft, writing – reviewing and editing. **Richard Evans‐Miller:** writing – original draft, writing – reviewing and editing. **Joe Perkins:** resources, writing – reviewing and editing. **Mohammed Malik:** methodology. **Martin Robinson:** reviewing and editing. **Gwendalyn Webb:** reviewing and editing. The authors take full responsibility for this article.

## Ethics Statement

This study received favorable ethics opinion on December 14, 2023 (reference number 1618441) from Nottingham Trent University: Social Sciences Research Ethics Committee.

## Conflicts of Interest

The authors declare no conflicts of interest.

## Supporting information


SUPPORTING INFORMATION S1.

## Data Availability

The data that support the findings of this study are openly available in Open Science Framework at https://doi.org/10.17605/OSF.IO/5NXUY [DOI link will be available after publication].
