# Supplementary material for: Dispositional Traits, Characteristic Adaptations, and Narrative Identity Reconstructions in Individuals With Depersonalization and Derealization
Source: J Pers. 2024 Oct 17;93(3):796–810. doi: 10.1111/jopy.12976 (PMC12053820; doi:10.1111/jopy.12976)
Supplement: Supplementary file 1 — SUPPORTING INFORMATION S1. [file JOPY-93-796-s001.docx]

**Personality, Characteristic Adaptations, and Narrative Identity Reconstructions in Individuals with Depersonalization and Derealization**

**Supplementary Material**

| **Table S1**  *Studies 1 and 2. Demographic information* | | | | |
| --- | --- | --- | --- | --- |
|  | **DPDR**  **(*n* = 160)** | **General**  **(*n* = 303)** | **Overall**  **(*N* = 463)** | **Test** |
| *Age* | 33.08 (13.95) | 39.74 (13.33) | 37.44 (13.90) | <.001 |
| Gender |  |  |  | < .001 |
| Female | 96 (60.00%) | 149 (49.17%) | 245 (52.92%) |  |
| Male | 48 (30.00%) | 153 (50.50%) | 201 (43.41%) |  |
| Other | 16 (10.00%) | 1 (0.33%) | 17 (3.67%) |  |
| *Highest qualifiication* |  |  |  | < .001 |
| A Levels | 33 (20.62%) | 45 (14.85%) | 78 (16.85%) |  |
| Degree | 7 (4.38%) | 117 (38.61%) | 124 (26.78%) |  |
| GCSE | 8 (5.00%) | 37 (12.21%) | 45 (9.72%) |  |
| NVQ | 71 (44.38%) | 44 (14.52%) | 115 (24.84%) |  |
| Postgraduate | 15 (9.38%) | 59 (19.47%) | 74 (15.98%) |  |
| Other | 26 (16.25%) | 1 (0.33%) | 27 (5.83%) |  |
| *Employment status* |  |  |  | < .001 |
| Carer | 0 (0.00%) | 1 (0.33%) | 1 (0.22%) |  |
| Full-time | 9 (5.62%) | 131 (43.23%) | 140 (30.24%) |  |
| Housewife/husband | 0 (0.00%) | 15 (4.95%) | 15 (3.24%) |  |
| Part-time | 20 (12.50%) | 51 (16.83%) | 71 (15.33%) |  |
| Retired | 20 (12.50%) | 19 (6.27%) | 39 (8.42%) |  |
| Self-employed | 30 (18.75%) | 29 (9.57%) | 59 (12.74%) |  |
| Student | 5 (3.12%) | 21 (6.93%) | 26 (5.62%) |  |
| Unemployed | 60 (37.50%) | 26 (8.58%) | 86 (18.57%) |  |
| Other | 16 (10.00%) | 9 (2.97%) | 25 (5.40%) |  |
| Missing | 0 (0%) | 1 (0.3%) | 1 (0.2%) |  |
| *Relationship status* |  |  |  | < .001 |
| Divorced | 3 (1.88%) | 14 (4.62%) | 17 (3.67%) |  |
| In a relationship | 7 (4.38%) | 82 (27.06%) | 89 (19.22%) |  |
| Married | 1 (0.62%) | 115 (37.95%) | 116 (25.05%) |  |
| Never married | 70 (43.75%) | 83 (27.39%) | 153 (33.05%) |  |
| Separated | 2 (1.25%) | 2 (0.66%) | 4 (0.86%) |  |
| Widowed | 33 (20.62%) | 5 (1.65%) | 38 (8.21%) |  |
| Other | 44 (27.50%) | 2 (0.66%) | 46 (9.94%) |  |

| **Table S2**  *Studies 1 and 2. Descriptive statistics* | | | | | | | | | | | |
| --- | --- | --- | --- | --- | --- | --- | --- | --- | --- | --- | --- |
|  | **DPDR (*n* = 160)** | | | | | **General (*n* = 303)** | | | | | |
|  | ***N*** | ***M*** | ***SD*** | **Skewness** | **Kurtosis** | | ***N*** | ***M*** | ***SD*** | **Skewness** | **Kurtosis** |
| Neuroticism | 160 | 3.39 | 0.63 | -0.39 | 0.11 | | 303 | 2.80 | 0.79 | 0.15 | -0.70 |
| Extraversion | 160 | 2.81 | 0.72 | 0.20 | -0.43 | | 303 | 2.93 | 0.74 | -0.09 | -0.30 |
| Openness | 160 | 3.64 | 0.57 | -0.31 | -0.49 | | 303 | 3.48 | 0.57 | -0.05 | -0.55 |
| Agreeableness | 160 | 4.06 | 0.52 | -0.73 | 0.39 | | 303 | 3.98 | 0.49 | -0.65 | 0.58 |
| Conscientiousness | 160 | 3.47 | 0.60 | 0.00 | -0.76 | | 303 | 3.71 | 0.64 | -0.44 | -0.26 |
| Negative Affect | 160 | 1.70 | 0.59 | -0.17 | -0.22 | | 303 | 1.08 | 0.75 | 0.23 | -0.99 |
| Detachment | 160 | 1.34 | 0.65 | 0.06 | -0.47 | | 303 | 0.92 | 0.72 | 0.55 | -0.46 |
| Antagonism | 160 | 0.52 | 0.49 | 1.12 | 0.97 | | 303 | 0.38 | 0.46 | 1.33 | 1.35 |
| Disinhibition | 160 | 0.79 | 0.71 | 0.88 | -0.03 | | 303 | 0.58 | 0.66 | 1.08 | 0.24 |
| Psychoticism | 160 | 1.78 | 0.61 | -0.18 | -0.49 | | 303 | 0.83 | 0.70 | 0.58 | -0.45 |
| Support | 160 | 3.34 | 1.24 | -0.23 | -0.65 | | 303 | 3.70 | 1.47 | -0.09 | -0.88 |
| Loneliness | 160 | 4.74 | 1.66 | -0.57 | -0.53 | | 303 | 3.66 | 1.76 | 0.17 | -0.98 |
| DPDR Frequency | 160 | 2.89 | 0.70 | 0.13 | -0.19 | |  |  |  |  |  |
| DPDR Duration | 160 | 3.55 | 1.22 | 0.22 | -0.63 | |  |  |  |  |  |

| **Table S3**  *Studies 1 and 2. Correlation matrix (Spearman) and omega reliability (MLR, 5,000 repetitions)* | | | | | | | | | | | | | | | | | |
| --- | --- | --- | --- | --- | --- | --- | --- | --- | --- | --- | --- | --- | --- | --- | --- | --- | --- |
|  | | 95% Confidence Interval | |  | | | | | | | | | | | | | |
|  | Omega | Lower | Upper |  |  |  |  |  |  |  |  |  |  |  |  |  |  |
| 1. Neuroticism | 0.76,0.88 | 0.69,0.86 | 0.82,0.9 |  | -.49*** | .05 | -.16** | -.56*** | .75*** | .56*** | .32*** | .40*** | .47*** | -.28*** | .52*** |  |  |
| 1. Extraversion | 0.82,0.86 | 0.77,0.84 | 0.86,0.89 | -.48*** |  | .12* | .05 | .34*** | -.34*** | -.55*** | .05 | -.03 | -.23*** | .35*** | -.38*** |  |  |
| 1. Openness | 0.52,0.68 | 0.4,0.62 | 0.64,0.74 | .08 | .21** |  | .16** | -.05 | .00 | -.10 | -.05 | .06 | .12* | -.06 | .01 |  |  |
| 1. Agreeableness | 0.76,0.75 | 0.69,0.68 | 0.82,0.81 | -.05 | -.08 | .03 |  | .32*** | -.15** | -.33*** | -.52*** | -.32*** | -.27*** | .19** | -.20*** |  |  |
| 1. Conscientiousness | 0.76,0.83 | 0.69,0.8 | 0.83,0.86 | -.32*** | .25** | .00 | .14 |  | -.48*** | -.49*** | -.42*** | -.59*** | -.58*** | .24*** | -.43*** |  |  |
| 1. Negative Affect | 0.55,0.81 | 0.4,0.78 | 0.69,0.85 | .56*** | -.13 | .08 | .15 | -.33*** |  | .56*** | .40*** | .46*** | .55*** | -.14* | .45*** |  |  |
| 1. Detachment | 0.65,0.81 | 0.56,0.77 | 0.73,0.85 | .29*** | -.46*** | -.33*** | -.12 | -.26*** | .12 |  | .40*** | .39*** | .58*** | -.32*** | .43*** |  |  |
| 1. Antagonism | 0.66,0.74 | 0.56,0.68 | 0.75,0.8 | .06 | .18* | .10 | -.50*** | -.25** | .09 | .01 |  | .49*** | .47*** | -.05 | .26*** |  |  |
| 1. Disinhibition | 0.82,0.86 | 0.76,0.83 | 0.87,0.89 | .19* | -.03 | -.07 | -.23** | -.68*** | .20** | .27*** | .33*** |  | .58*** | -.03 | .25*** |  |  |
| 1. Psychoticism | 0.71,0.83 | 0.63,0.8 | 0.78,0.86 | .19* | -.04 | .18* | .06 | -.32*** | .33*** | .19* | .19* | .34*** |  | -.10 | .35*** |  |  |
| 1. Support | 0.9,0.94 | 0.87,0.92 | 0.93,0.95 | -.15 | .21** | .20* | .14 | .10 | -.02 | -.25** | .01 | -.08 | .05 |  | -.36*** |  |  |
| 1. Loneliness | 0.88,0.93 | 0.84,0.91 | 0.92,0.94 | .37*** | -.31*** | -.06 | -.09 | -.19* | .29*** | .31*** | .13 | .15 | .13 | -.36*** |  |  |  |
| 1. DPDR Frequency | 0.93,NA | 0.91,NA | 0.95,NA | .16* | -.13 | -.17* | .05 | -.11 | .24** | .41*** | .11 | .21** | .40*** | -.13 | .30*** |  |  |
| 1. DPDR Duration | 0.95,NA | 0.93,NA | 0.96,NA | .11 | -.09 | -.18* | .01 | -.15 | .22** | .32*** | .14 | .14 | .33*** | -.12 | .25** | .82*** |  |
| Note. DPDR sample (*n* = 160) in the lower triangle, general population sample (*n* = 303) in the upper triangle. *** indicates significance at Alpha = .001, ** at .01, * at .05. Omega values represent estimates for, respectively, the DPDR and the general population samples. | | | | | | | | | | | | | | | | | |

| **Table S4**  *Study 1. Weight matrix from independent graphical models* | | | | | | | | | | | |
| --- | --- | --- | --- | --- | --- | --- | --- | --- | --- | --- | --- |
|  | **1.** | **2.** | **3.** | **4.** | **5.** | **6.** | **7.** | **8.** | **9.** | **10.** | **11.** |
| 1. Neuroticism |  |  |  |  |  | .49 | .07 |  |  |  |  |
| 1. Extraversion |  |  | .04 |  | .05 |  |  | .15 | .07 |  | .15 |
| 1. Openness |  |  |  | .08 |  |  |  |  |  | .06 |  |
| 1. Agreeableness |  |  |  |  | .05 |  |  |  |  |  | .04 |
| 1. Conscientiousness |  | .01 |  |  |  |  |  |  |  |  |  |
| 1. Negative Affect | .33 |  |  |  |  |  | .12 | .08 | .08 | .15 |  |
| 1. Detachment | .01 |  |  |  |  |  |  | .11 |  | .25 |  |
| 1. Antagonism |  |  |  |  |  |  |  |  | .19 | .11 |  |
| 1. Disinhibition |  |  |  |  |  |  | .05 | .11 |  | .23 |  |
| 1. Psychoticism |  |  |  |  |  | .11 |  |  | .09 |  |  |
| 1. Support |  |  |  |  |  |  |  |  |  |  |  |
| 1. Loneliness | .13 |  |  |  |  | .03 | .08 |  |  |  |  |
| Note. DPDR sample (*n* = 160) in the lower triangle, general population sample (*n* = 303) in the upper triangle. Values indicated Spearman’s correlations after EBICglasso regularization. | | | | | | | | | | | |

| **Table S5**  *Study 1. Network comparison test (edges; N_DPDR_ = 160, N_Controls_ = 303)* | | | | |
| --- | --- | --- | --- | --- |
| **Var1** | **Var2** | **Test statistic (*E*)** | ***p*** | ***p_FDR_*** |
| Neuroticism | Extraversion | .061 | .435 | 1.000 |
| Neuroticism | Openness | .000 | 1.000 | 1.000 |
| Extraversion | Openness | .035 | .734 | 1.000 |
| Neuroticism | Agreeableness | .000 | 1.000 | 1.000 |
| Extraversion | Agreeableness | .000 | 1.000 | 1.000 |
| Openness | Agreeableness | .084 | .391 | 1.000 |
| Neuroticism | Conscientiousness | .118 | .110 | .807 |
| Extraversion | Conscientiousness | .043 | .726 | 1.000 |
| Openness | Conscientiousness | .000 | 1.000 | 1.000 |
| Agreeableness | Conscientiousness | .052 | .487 | 1.000 |
| Neuroticism | Negative Affect | .158 | .034 | .739 |
| Extraversion | Negative Affect | .000 | 1.000 | 1.000 |
| Openness | Negative Affect | .000 | 1.000 | 1.000 |
| Agreeableness | Negative Affect | .000 | 1.000 | 1.000 |
| Conscientiousness | Negative Affect | .071 | .152 | .835 |
| Neuroticism | Detachment | .065 | .387 | 1.000 |
| Extraversion | Detachment | .095 | .291 | 1.000 |
| Openness | Detachment | .112 | .402 | 1.000 |
| Agreeableness | Detachment | .075 | .415 | 1.000 |
| Conscientiousness | Detachment | .025 | .802 | 1.000 |
| Negative Affect | Detachment | .117 | .071 | .807 |
| Neuroticism | Antagonism | .000 | 1.000 | 1.000 |
| Extraversion | Antagonism | .154 | .246 | 1.000 |
| Openness | Antagonism | .000 | 1.000 | 1.000 |
| Agreeableness | Antagonism | .045 | .633 | 1.000 |
| Conscientiousness | Antagonism | .048 | .565 | 1.000 |
| Negative Affect | Antagonism | .080 | .371 | 1.000 |
| Detachment | Antagonism | .109 | .151 | .835 |
| Neuroticism | Disinhibition | .000 | 1.000 | 1.000 |
| Extraversion | Disinhibition | .072 | .655 | 1.000 |
| Openness | Disinhibition | .000 | 1.000 | 1.000 |
| Agreeableness | Disinhibition | .019 | .832 | 1.000 |
| Conscientiousness | Disinhibition | .180 | .030 | .739 |
| Negative Affect | Disinhibition | .082 | .126 | .834 |
| Detachment | Disinhibition | .052 | .560 | 1.000 |
| Antagonism | Disinhibition | .077 | .312 | 1.000 |
| Neuroticism | Psychoticism | .000 | 1.000 | 1.000 |
| Extraversion | Psychoticism | .000 | 1.000 | 1.000 |
| Openness | Psychoticism | .063 | .574 | 1.000 |
| Agreeableness | Psychoticism | .000 | 1.000 | 1.000 |
| Conscientiousness | Psychoticism | .135 | .088 | .807 |
| Negative Affect | Psychoticism | .036 | .615 | 1.000 |
| Detachment | Psychoticism | .249 | .005 | .356 |
| Antagonism | Psychoticism | .112 | .173 | .880 |
| Disinhibition | Psychoticism | .136 | .099 | .807 |
| Neuroticism | Support | .000 | 1.000 | 1.000 |
| Extraversion | Support | .150 | .094 | .807 |
| Openness | Support | .000 | 1.000 | 1.000 |
| Agreeableness | Support | .042 | .712 | 1.000 |
| Conscientiousness | Support | .005 | .891 | 1.000 |
| Negative Affect | Support | .000 | 1.000 | 1.000 |
| Detachment | Support | .023 | .781 | 1.000 |
| Antagonism | Support | .000 | 1.000 | 1.000 |
| Disinhibition | Support | .000 | 1.000 | 1.000 |
| Psychoticism | Support | .000 | 1.000 | 1.000 |
| Neuroticism | Loneliness | .053 | .497 | 1.000 |
| Extraversion | Loneliness | .019 | .835 | 1.000 |
| Openness | Loneliness | .000 | 1.000 | 1.000 |
| Agreeableness | Loneliness | .000 | 1.000 | 1.000 |
| Conscientiousness | Loneliness | .121 | .062 | .807 |
| Negative Affect | Loneliness | .023 | .778 | 1.000 |
| Detachment | Loneliness | .014 | .857 | 1.000 |
| Antagonism | Loneliness | .013 | .876 | 1.000 |
| Disinhibition | Loneliness | .000 | 1.000 | 1.000 |
| Psychoticism | Loneliness | .000 | 1.000 | 1.000 |
| Support | Loneliness | .030 | .743 | 1.000 |

| **Table S6**  *Study 1. Network comparison test (expected influence centrality; N_DPDR_ = 160, N_Controls_ = 303)* | | | |
| --- | --- | --- | --- |
|  | ***p*** | ***p_FDR_*** |  |
| Neuroticism | .279 | .419 |  |
| Extraversion | .253 | .419 |  |
| Openness | .162 | .350 |  |
| Agreeableness | .860 | .860 |  |
| Conscientiousness | .682 | .744 |  |
| Negative Affect | .015 | .090 |  |
| Detachment | .175 | .350 |  |
| Antagonism | .088 | .350 |  |
| Disinhibition | .014 | .090 |  |
| Psychoticism | .124 | .350 |  |
| Support | .456 | .608 |  |
| Loneliness | .674 | .744 |  |

| **Table S7**  *Study 3. Demographic characteristic of participants and other related information (N = 14*)* | | | | | | | |
| --- | --- | --- | --- | --- | --- | --- | --- |
| **Pseudonym** | **Age** | **Gender** | **Education** | **Employment** | **Relationship** | **Formally diagnosed** | **Ever been in treatment** |
| Maria | 24 | Female | NVQ | Other | Other | Yes | Yes |
| Beth | 30 | Female | GCSE | Self-employed | Never married | No | Yes |
| James | 35 | Male | A-Levels | Unemployed | Never married | Yes | Yes |
| Lara | 22 | Other | NVQ | Self-employed | Other | No | Yes |
| Fred | 27 | Other | A-Levels | Full-time | Never married | Yes | No |
| Ola | 18 | Other | Other | Part-time | Never married | No | Yes |
| Richard | 43 | Male | A-Levels | Unemployed | Other | Yes | Yes |
| Jack | 38 | Male | GCSE | Unemployed | Never married | Yes | Yes |
| Dan | 63 | Male | NVQ | Other | Widowed | No | Yes |
| Rose | 39 | Female | NVQ | Self-employed | Never married | No | Yes |
| Liz | 49 | Female | A-Levels | Unemployed | Widowed | No | Yes |
| April | 21 | Other | Other | Unemployed | Never married | Yes | Yes |
| Daisy | 29 | Female | NVQ | Other | Never married | Yes | Yes |
| Sara | 24 | Female | Other | Part-time | Never married | Yes | Yes |
| *Note.* *Five participants could not retrieve their unique identifier at the time of the interview, making their demographics unknown. | | | | | | | |

| **Table S8**  *Study 3. Summary of themes and sub-themes (N = 19)* | |
| --- | --- |
| **Theme** | **Sub-theme** |
| 1. “How it started and what happened next”: DPDR onset as a major life turning point and contaminator. | 1. DPDR onset as a life turning point and contaminator. |
|  | 2. The challenge of making sense of altered perceptual boundaries. |
|  | 3. Difficulties with describing, characterizing, and communicating symptoms. |
|  | 4. Existentialist thinking and rumination. |
|  | 5. Failures with healthcare. |
| 2. “Why me?” Meaning-making, exploratory narratives associated with the lived experience of DPDR. | 1. Traumatic antecedents. |
|  | 2. Feeling like losing control. |
|  | 3. Alternance of Derealization and Depersonalization. |
| 3. “The more you think about it, the worse it gets”: Coping strategies, tentative redemptions, and failed resolutions. | 1. Management of short-term anxiety, rumination, and fear. |
|  | 2. Long-term coping strategies and approaches to recovery. |
|  | 3. Distraction and diversion as coping strategies. |
|  | 4. Finding out about the condition. |
|  | 5. Tentative redemptions and unsuccessful therapies. |
| 4. “It’s kind of hindered things for me”: Living with DPDR, disrupted agency and communion. | 1. Impact on interpersonal self-efficacy. |
|  | 2. Negative self-identifications with DPDR. |
|  | 3. Beliefs about the chronic and irreversible nature of DPDR. |
|  | 4. Talking with other people with DPDR does not help. |
|  | 5. The role of social/emotional support offered by close ones |


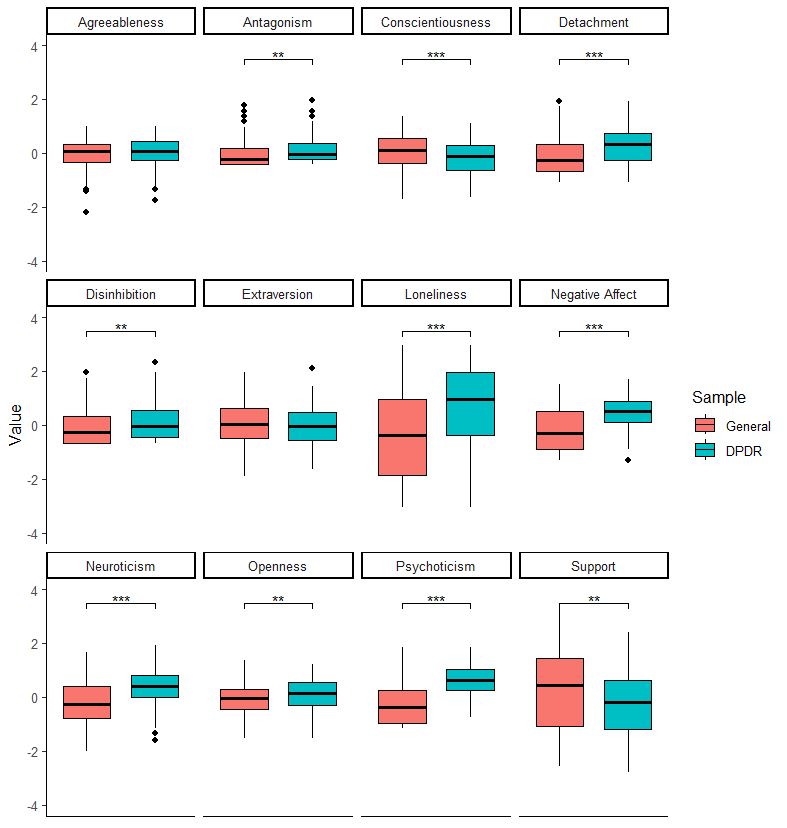


Figure FS1. Study 1. Independent samples mean differences (N_DPDR_ = 160, N_Controls_ = 303). For each variable, values are mean-centered.


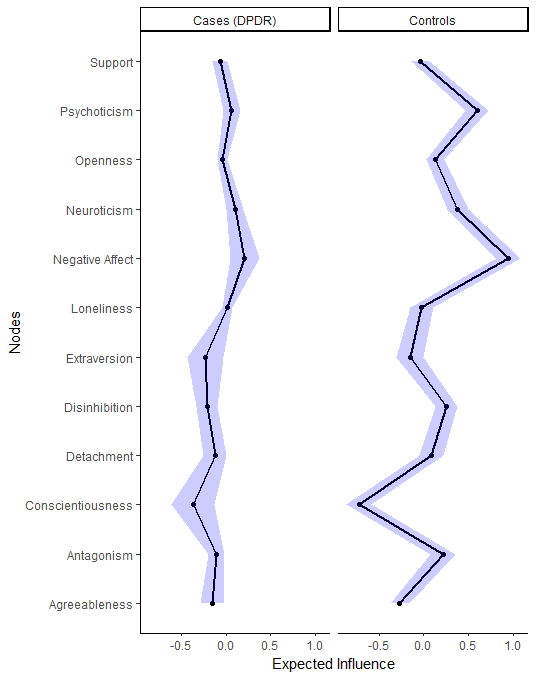


Figure FS2. Study 1. Centrality point estimates (mean-centered) from bootstrapping (i.e., obtained by dropping 5% to 75% of cases in steps of 5%, 1,000 samples examined at each stage) (N_DPDR_ = 160, N_Controls_ = 303). Shaded areas represent estimates ± standard errors obtained via bootstrapping.


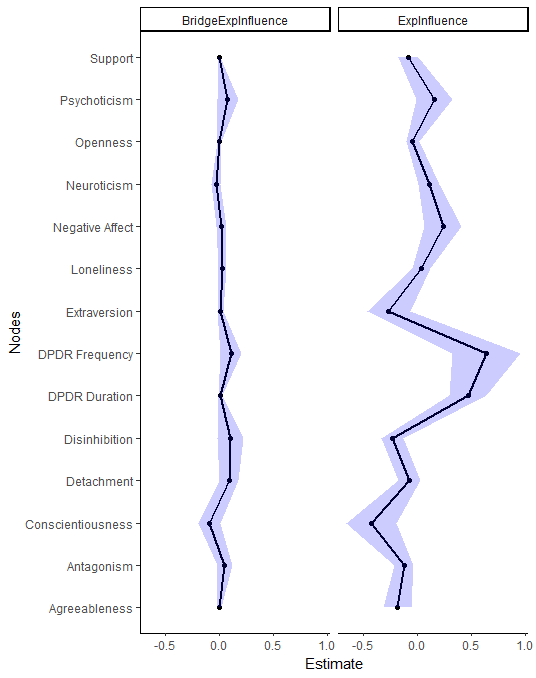


Figure FS3. Study 2. Centrality point estimates (mean-centered) from bootstrapping (i.e., obtained by dropping 5% to 75% of cases in steps of 5%, 1,000 samples examined at each stage); (N_DPDR_ = 160). Shaded areas represent estimates ± standard errors obtained via bootstrapping.
